# Modelling COVID-19 transmission in supermarkets using an agent-based model

**DOI:** 10.1371/journal.pone.0249821

**Published:** 2021-04-09

**Authors:** Fabian Ying, Neave O’Clery

**Affiliations:** 1 Quantitative Research, G-Research, London, United Kingdom; 2 Centre for Advanced Spatial Analysis, UCL, London, United Kingdom; University of Zambia, ZAMBIA

## Abstract

Since the outbreak of COVID-19 in early March 2020, supermarkets around the world have implemented different policies to reduce the virus transmission in stores to protect both customers and staff, such as restricting the maximum number of customers in a store, changes to the store layout, or enforcing a mandatory face covering policy. To quantitatively assess these mitigation methods, we formulate an agent-based model of customer movement in a supermarket (which we represent by a network) with a simple virus transmission model based on the amount of time a customer spends in close proximity to infectious customers (which we call the exposure time). We apply our model to synthetic store and shopping data to show how one can use our model to estimate exposure time and thereby the number of infections due to human-to-human contact in stores and how to model different store interventions. The source code is openly available under https://github.com/fabianying/covid19-supermarket-abm. We encourage retailers to use the model to find the most effective store policies that reduce virus transmission in stores and thereby protect both customers and staff.

## Introduction

As the main provider of food and essential goods, supermarkets remained open in many countries throughout the COVID-19 pandemic in 2020, while the majority of other businesses (such as general retail stores) shut down during periods of government-mandated lockdowns [1, 2]. Supermarkets represent one of the main hubs where a large number of people mix indoors throughout the pandemic and are thus a potential risk area where the virus SARS-CoV-2, which causes COVID-19, may be transmitted. It is therefore vital to find safe ways for customers to shop and minimize virus transmission. Models for customer dynamics and virus transmission are useful towards that goal, as they can be used to estimate the infection risk and assess how different interventions affect the risk. In this article, we propose an agent-based model for customer dynamics which we use to estimate the total amount of *exposure time*, which we define as the total amount of time that customers are in close proximity to infected customers. Using a simple virus transmission model, we estimate the number of infections from exposure time. We thereby ignore fomite and airborne transmission and only consider infections due to respiratory droplet transmission, which the World Health Organisation (WHO) and the US Centre for Disease Control and Prevention (CDC) consider the main mode of transmission of COVID-19 [3, 4]. We apply this model to synthetic data and how to model the following interventions:

- Restricting the maximum number of customers in the store,
- Reducing the rate at which customers enter the store,
- Implementing face mask policy, and
- One-way aisle store layout.

These and other interventions have been used or recommended in supermarkets in the UK and the US [5–7], among other countries.

## Related work

Existing work for indoor COVID-19 transmission mainly consider airborne transmission rather than respiratory droplet transmission, with a focus on how ventilation and other environmental factors affect airborne transmission risk. The models are typically either Computational Fluid Dynamics (CFD) models [8–10], airflow models based on partial differential equations (PDEs) [11], or models that make a well-mixed room (WMR) assumption, where virus-carrying aerosols are instantaneously evenly distributed across a room [12–15]. CFD models take a long time to run and are typically intractable for large-scale simulations, particularly in situations where there are many moving objects (such as customers in a store). The WMR room models generally only consider a single room and do not take into account the geometry of a store. PDEs models sit in between CFD and WMR models, allowing for more complicated geometry to be taken into account while being computationally tractable. However, none of these models take close contacts due to customer mobility and the resulting droplet transmission risk into account. Furthermore, existing work has not studied how changes in store policy or layout affect infection risk.

In this work, our focus is on respiratory droplet transmission due to customers coming into close contact with one another. We use an agent-based model as it allows us to take into account the non-linearities of the system due to customer mobility and the layout of the store, while being computationally cheap enough to run a large number of simulations without the need to solve any differential equations, as we ignore airborne transmission. Plata et al. [16] proposed a similar model to ours, but on a higher spatial resolution than what we consider. However, their model is computationally more expensive, and their code is not openly available.

## Materials and methods

### Store graph

**Fig 1:**
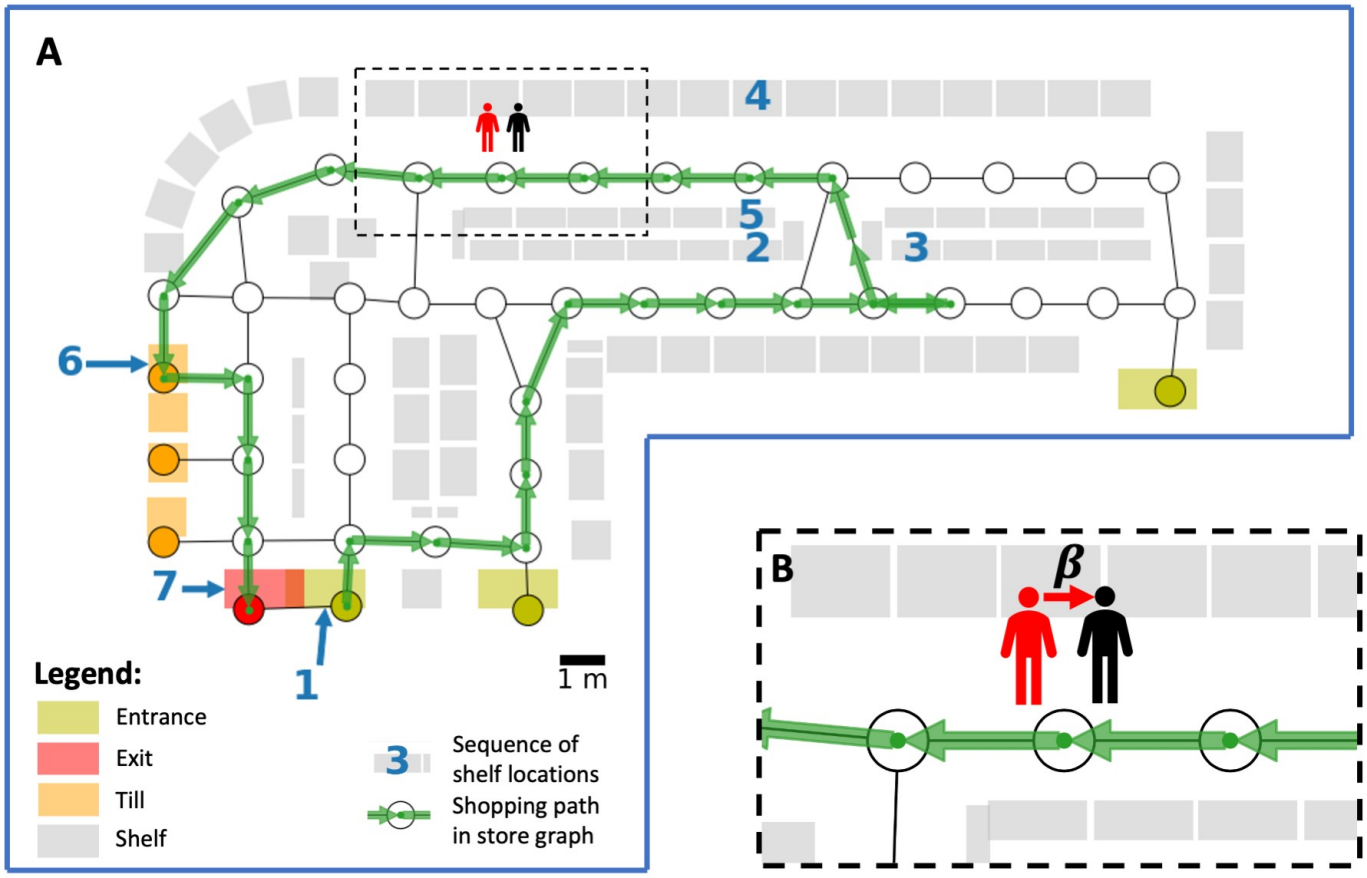
Overview of agent-based model. **(A)** A network representation of a small supermarket/convenience store with an example shopping path (in green). We generate each shopping path from a sequence of shelf locations (in blue), which correspond to the shelves from a customer picks up their items during a visit and the entrance and the tills. In this example, the customer picks up *K* = 4 items at the shelves marked in blue with 2, 3, 4, and 5. **(B)** Virus transmission model. A susceptible customer (in black) becomes infected at rate *β* whenever they are in the same zone as an infectious customer (in red).

We represent a store as a network (called a *store graph*), in which nodes represent zones and edges connect contiguous zones. We create a store graph from a synthetic store layout following a similar procedure as in [17, 18]. Zones are approximately 2m by 2m and we specify a number of entrance, till, and exit nodes (see Fig 1A). We choose a network representation of a store (instead of modelling on the underlying space directly, see e.g., [16]) for ease of simulation, as it significantly reduces the complexity of the model.

### Agent-based model

Our agent-based model has two components: a customer mobility model and a virus transmission model. The first component is the customer mobility model for how customers arrive at the store and move. The second component is a model for how the virus transmits in the supermarket.

#### Customer mobility model

In our agent-based model, customers arrive the store according to a Poisson process with constant rate λ. Each customer starts at a random entrance node (chosen uniformly at random from all entrance nodes) and is assigned a shopping path, chosen uniformly at random from all shopping paths from a synthetic data set or an empirical data set (if available). (In this article, we only consider synthetic data sets, as no empirical ones were available to us.) Each shopping path is a path in the store graph, representing the route that a customer takes in the store. Two consecutive nodes in the shopping path may be identical. This case occurs when a customer picks up one or more items in the zone. A customer traverses the store graph according to its assigned shopping path. At each node, a customer waits a random time *T*, which is exponentially distributed with mean *τ* (independent of other waiting times), before traversing to the next node in the shopping path (or staying at the same node, if it is the next node). Once a customer arrives at the final node (which is the exit node) in its shopping path, the customer stays *T* seconds on the node (with *T* again exponentially distributed with mean *τ*) and is then removed from the system. At the beginning of each simulation, the store is empty and customers arrive in the store over a period of *H* hours (corresponding to length of the opening hours of the store). After *H* hours, no new customers arrive and the simulation stops once the last customer leaves the store.

#### Virus transmission model

Customer are either susceptible or infectious when they enter the store. Each customer that arrives to the store is infectious with independent probability *p*_*I*_ (corresponding to the proportion of infectious customers) and is otherwise susceptible. In our infection mechanism, we assume susceptible customers become infected proportional to the time they spent with infectious customers. We assume that the main mode of transmission is direct transmission via respiratory droplets and neglect airborne transmission and fomite transmission. More formally, we define the *exposure time* *E*_*s*_ for each susceptible customer *s* as follows. We define the *individual exposure time* $E_{s}^{\left(i\right)}$ to an infectious customer *i* as the total time that customer *s* was in the same zone as an infectious customer *i* during the shopping trip of *s*. Then $E_{s}=\sum_{i}E_{s}^{\left(i\right)}$ is the sum of the individual exposure times. We call a susceptible customer *exposed* if they have positive exposure time. Each exposed customer becomes infected after the shopping trip with probability min(*βE*_*s*_, 1) for some transmission rate *β* > 0. In other words, we model the infection probability of an exposed customer as a linear function of the exposure time with infectious customers. In reality, the infection probability function may take some other form (e.g., a logistic function), but for simplicity and due to lack of validated alternative models, we choose a linear function. We illustrate the virus transmission model in see Fig 1B.

Our model has two main outputs: The total number of infections and the chance of infection for a given simulation period. These two quantities depend linearly on the *total exposure time* (defined as the sum of exposure times) and the mean exposure time per susceptible customer.

### Data

We use a synthetically-created store layout and shopping path data with 10^6^ paths. The (synthetic) store is a small store with around 80 shelves, four tills, three entrances, and one exit (see Fig 1). We synthetically generate 10^6^ shopping paths as follows. For each path, we first sample the number *K* of items that the customer picks up during their shop. We choose a log-normal distribution for *K* where the underlying normal distribution has mean *μ* and standard deviation *σ* [19]. We then choose a sequence of locations (*s*_1_, …, *s*_*K*+3_), where *s*_1_ is a random entrance, *s*_2_, …, *s*_*K*+1_ are random shelves (chosen uniformly at random from all shelves with replacement), *s*_*K*+2_ is a random till, and *s*_*K*+3_ is the exit. The locations *s*_2_, …, *s*_*K*+1_ represent the *K* shelves where a customer buys their items in their shopping trip. We map each location *s*_*i*_ to the corresponding node *v*_*i*_ in the store graph that contains the shelf, entrance, till, or exit location. We then generate the shopping path from the node sequence (*v*_1_, …, *v*_*K*+3_) by choosing a shortest path in the store graph that visits each of these nodes in sequence. We show an example sequence of locations and its corresponding shopping path in Fig 1A.

### Parameter values

In our simulations, we set the default arrival rate to be 2.55 customers per minute. This is based on the mean number of baskets per store over a 91-day period across 17 UK supermarkets as reported in [17] and the typical UK store opening period of 14 hours [20]. (In [17], Ying *et al*. used a data set of 13672 mean baskets per store over 91 days, representing a sample of 7% of the total number of baskets. Therefore, the customer arrival rate is 13672/(0.07 × 91 × 14 × 60) = 2.55 customers per minute over this period.) We assume that each basket corresponds to a single customer (rather than groups of customers). At the time of writing, many UK supermarkets advise or restrict customers to shop alone [21].

We choose *μ* = 0.07 and *σ* = 0.76 for the parameters of the log-normal distribution for sampling the item basket size *K*. These values correspond to the ones reported in [19] for small supermarkets and convenience stores. We infer the mean wait time *τ* at each node from the mean shopping time of 5.95 minutes for small stores as reported in [19] and the mean shopping path length of 29.66 nodes based on the synthetic data described above. This yields a mean wait time of *τ* equals to 5.95/29.66 = 0.2 minutes per node. Note that data on the mean shopping length and the arrival rate is from before the COVID-19 pandemic; these values may differ during a pandemic due to a change in customer shopping behaviour.

We use *p*_*I*_ = 1.87% as the proportion of infectious customers based on data reported from UK’s Office for National Statistics (ONS) Infection Survey from January 2021 [22]. In this survey, a random sample of the population is tested for COVID-19 to estimate the overall proportion of people who have COVID-19 at that particular point in time.

The transmission rate *β* is much harder to estimate than the previous parameters because very little data exists. In [23], the mean probability of transmission per contact is estimated to be 2.11 × 10^−8^. However, they do not specify the duration of a contact. We use this parameter and assume that the mean contact duration is 15 minutes to obtain a rate of transmission to be *β* = 2.11 × 10^−8^/15 = 1.41 × 10^−9^ per minute. (We assume that the probability of transmission is proportional to the contact duration).

We summarize the parameter values that we use in Table 1.

**Table 1:** Parameter values in our agent-based model. We list uncertainty bounds, when available.

Parameter	Default value	Reference/Assumption
Parameters (*μ*, *σ*) of log-normal distribution for basket size	(0.07, 0.76)	[19]
Arrival rate (λ)	2.55 customer/min	[17]
Mean wait time at each node (*τ*)	0.2 min	inferred from [19]
Percentage of infectious customers (*p*_*I*_)	1.87% 95% conf. interval: (1.79%, 1.95%)	[22]
Transmission rate (*β*)	1.41 × 10^−9^ per min 95% conf. interval: (1.15, 1.66) × 10^−9^	inferred from [23] (assuming mean contact duration of 15 minutes)
Length of opening hours (*H*)	14 hours	[20]
Store layout	fixed	Layout of synthetic store

### Analysis environment

We implemented our agent-based model in Python 3.6 using SimPy 4 [24]. We ran our simulations on an Amazon Web Service cluster (ml.c5.4xlarge with 32 GB RAM and 16 vCPU) [25], although all simulations can be performed on a standard desktop computer.

## Results and discussion

We demonstrate in this section how to use our model, what metrics we can record in it, and compare different interventions on our synthetic store.

**Fig 2:**
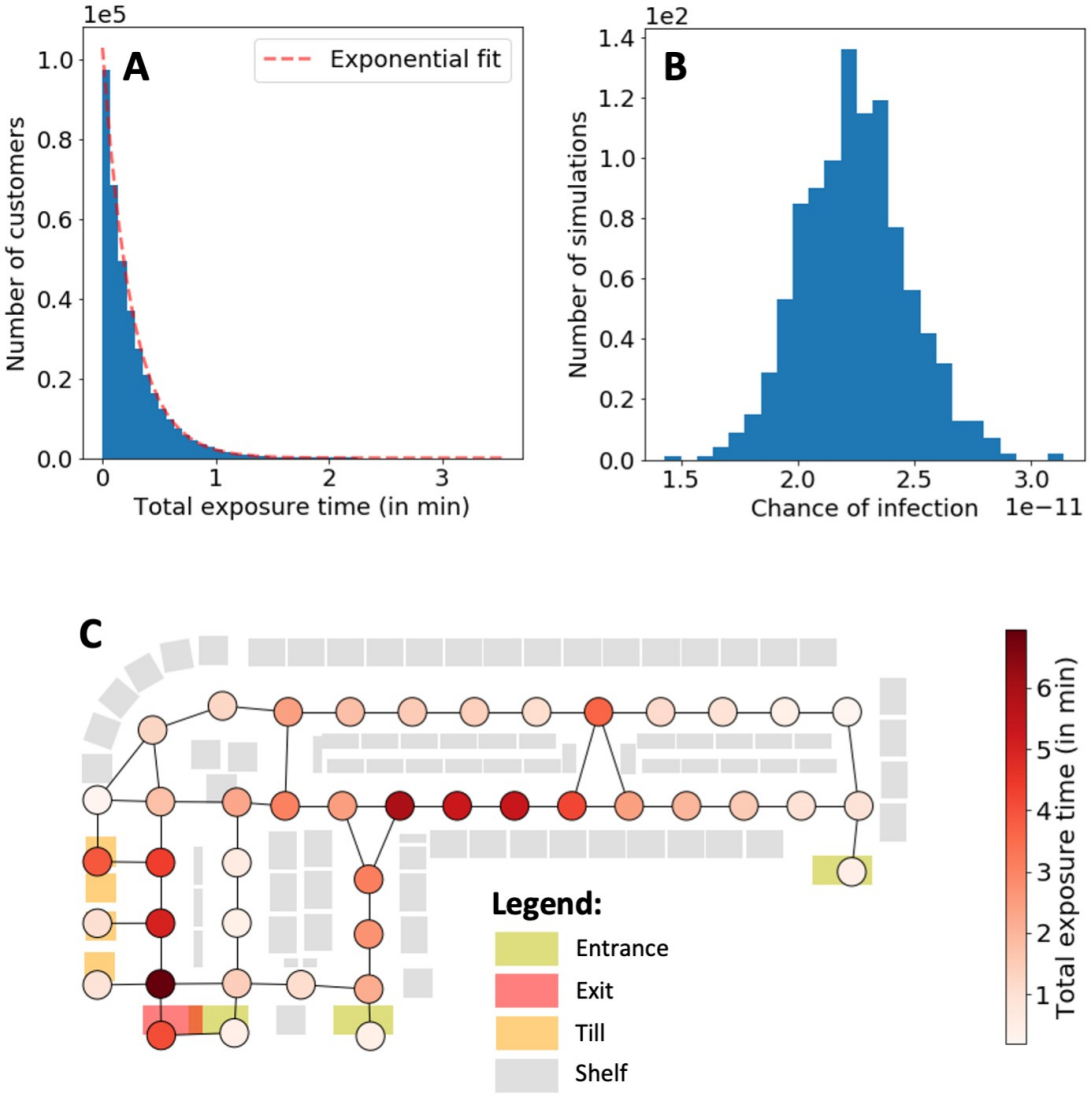
Exposure times and chance of infection from 1000 simulations. **(A)** Histogram of total customer exposure time for all exposed customers (i.e., susceptible customers with positive exposure time) across 1000 simulations. The distribution can be approximated by an exponential distribution. **(B)** Histogram of chance of infection per susceptible customer across 1000 simulations. **(C)** Total exposure time per node. Nodes in the centre and near the tills of the store show significantly higher amount of exposure time than others.

Using the default parameters listed in Table 1, we perform 1000 simulations, each simulating a day in our synthetic store. Customers stay on average 5.97 minutes in the store, with on average 14.96 customers present in the store at any given time. With *p*_*I*_ = 1.87% infected customers, the total exposure time (i.e., the exposure time summed over all susceptible customers) is on average 94.98 min per day. Multiplying this with *β* = 1.41 × 10^−9^ infections per minute of exposure time, we estimate an average of 1.34 × 10^−7^ infections per day. Each susceptible customer is exposed 0.0452 minutes (or about 2.71 seconds) on average per visit to an infected customer and the estimated a chance of infection is 6.37 × 10^−11^. The mean individual exposure time is dominated by a majority of susceptible customers that were not exposed (i.e., who had no exposure with infected customers); only 17.69% of susceptible customers had any exposure. Among the exposed customers, the exposure time appears to be exponentially distributed with a mean exposure time of 0.26 minutes. We note that the majority of exposed customers have a short exposure time of less than 1 minute (see Fig 2A). The longest exposure time of a customer in our simulations is 3.5 minutes. Therefore, if we used an alternative virus transmission model where an infection can only occur after 15 minutes of cumulative exposure time, for example, we would not record any infections in our simulations. The CDC and many other national health agencies at the time of writing [26–28] define a close contact (within 2 metres) to occur when there is 15 minutes of cumulative exposure. However, infections may still occur when a customer has additional exposure in a different setting (e.g., in another shop) during a 24-hour window [27, 29]. Therefore, reducing exposure time is still desirable, even if the maximum exposure time is short in a supermarket.

The chance of infection per susceptible customer does not show large deviations across simulations (see Fig 2B). We list all metrics that we record in our simulations in Table 2.

**Table 2:** Simulation results. We show the mean and standard deviation of each metric across 1000 simulations.

Metric	Mean	Standard deviation
Number of customers	2143	46.76
Number of susceptible customers	2103	46.52
Number of infected customers	39.28	5.44
Mean number of customers in store	14.96	0.45
Mean shopping time (in min)	5.97	0.06
Total exposure time (in min)	94.98	15.38
Exposure time per sus. customer (in min)	0.0452	0.0073
Exposure time per exposed customer (in min)	0.26	0.27
Percentage of sus. customers with exposure	17.69%	2.40%
Number of infections	1.34 × 10^−7^	2.17 × 10^−8^
Chance of infection per sus. customer	6.37 × 10^−11^	1.03 × 10^−11^

Our models also allow us to record the total exposure time for each node *v* (defined as the sum of the individual exposure times that occurred on *v*). We see in Fig 2C that near the tills and centre of the store shows the highest amount of exposure time, thereby revealing mobility flow bottlenecks in the store that may be mitigated by changing to a different layout.

### Restricting the maximum number of customers in store

**Fig 3:**
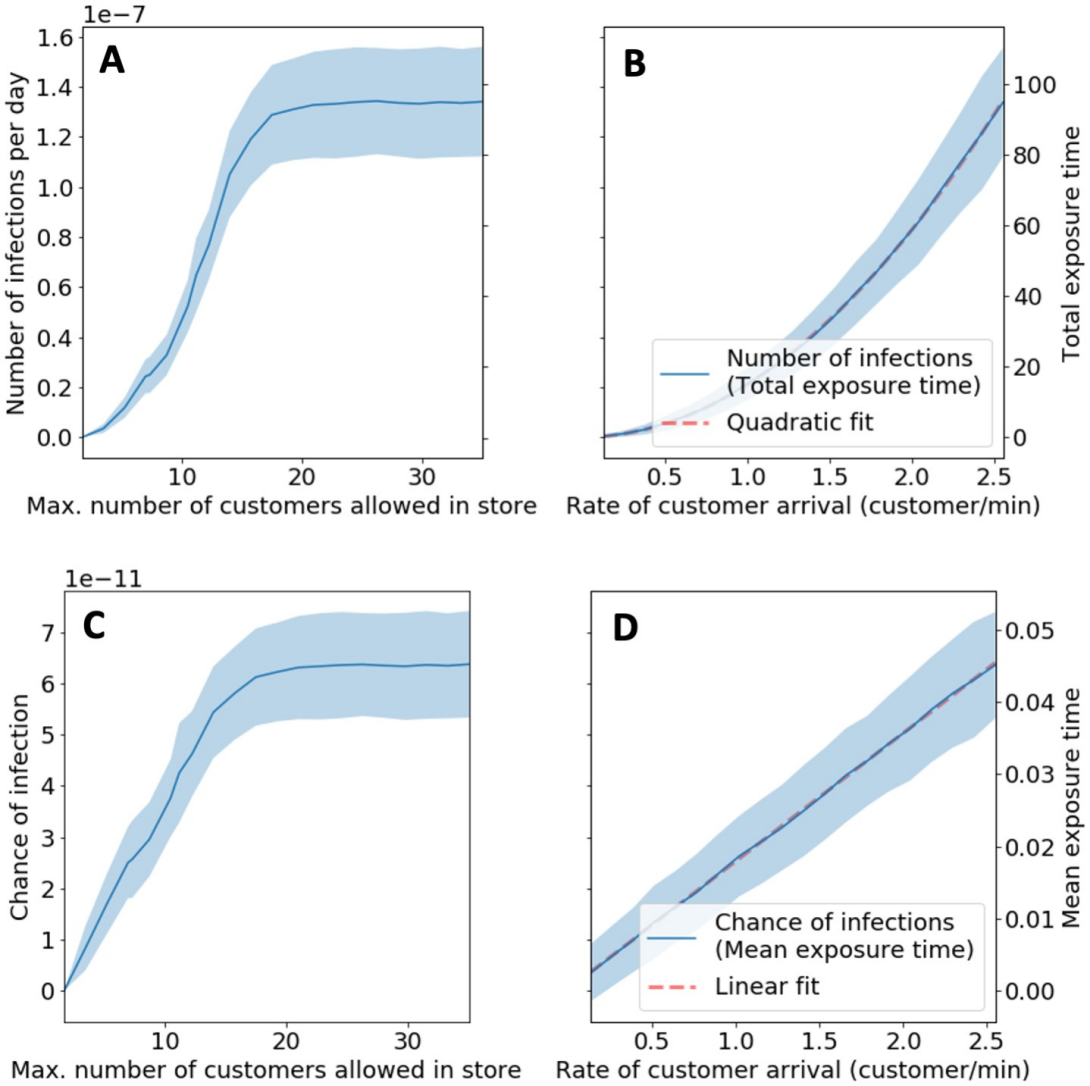
Change in number of infections and chance of infection by reducing maximum number of customers in store or arrival rate. **(A + B)** Mean number of infections (with the shaded area showing the standard deviation) as a function of maximum number *C*_max_ of customers and customer arrival rate λ (respectively). As the number of infections is a linear function of the total exposure time, we also show the total exposure time on the right vertical axis. **(C + D)** Mean chance of infection for each susceptible customer (with the shaded area showing the standard deviation) as a function of maximum number *C*_max_ of customers and customer arrival rate λ (respectively). As the chance of infection is a linear function of the mean exposure time (per susceptible customer), we also show the mean exposure time on the right vertical axis. In subfigures (A) and (C), the mean number of infections and mean chance of infection plateaus, as the number of customers typically does not exceed 20 in our simulations.

In line with UK government guidelines [30], many stores restrict the maximum number *C*_max_ of customers in a store. We can add this restriction to our model by simulating a queue outside of the store, where customers queue up if we have *C*_max_ or more customers in the store. Customers from the queue only enter the store when the number of customers in the store is below *C*_max_. In our model, the estimated chance of infection and number of infections also decreases significantly when decreasing the maximum number of customers in the store (see Fig 3A+3C). We also note that the mean number of infections plateaus as we increase the *C*_max_ beyond 20, as the number of customers typically does not exceed 20 in our simulations.

### Reducing customer arrival rate

Another way of reducing the number of customers in the store is to restrict the rate at which customers enter the store. We can incorporate this in our model by varying the arrival rate λ. We see that the chance of infection increases linearly with λ while the number of infections increases quadratically (see Fig 3B+3D). The linear and quadratic scaling are not unsurprising: The number of customers (both infectious customers and susceptible customers) in the store increases linearly with the arrival rate. Therefore, we expect the exposure time (and hence the chance of infection) for each susceptible customer to increase linearly with the arrival rate λ. For the quadratic scaling of the number of infections, note that the total number of infections is the product of the number of susceptible customers and the chance of infection. Both quantities scale linearly with the arrival rate, which gives a quadratic scaling for the number of infections.

Note that restricting *C*_max_ is equivalent to reducing the arrival rate λ, as both methods reduce the rate at which customers enter the store.

### Face masks

Similar to [31], we model the implementation of a face mask policy via a reduction in the transmission rate. For example [32], estimated the relative (transmission) risk reduction to be *RRR* = 0.17. We incorporate this by multiplying *β* with *RRR*, reducing the number of infections and chance of infection by the same factor: the number of infections decreases from 1.34 × 10^−7^ to 2.28 × 10^−8^; the chance of infection decreases from 6.37 × 10^−11^ to 1.08 × 10^−11^.

### One-way aisle layout

**Fig 4:**
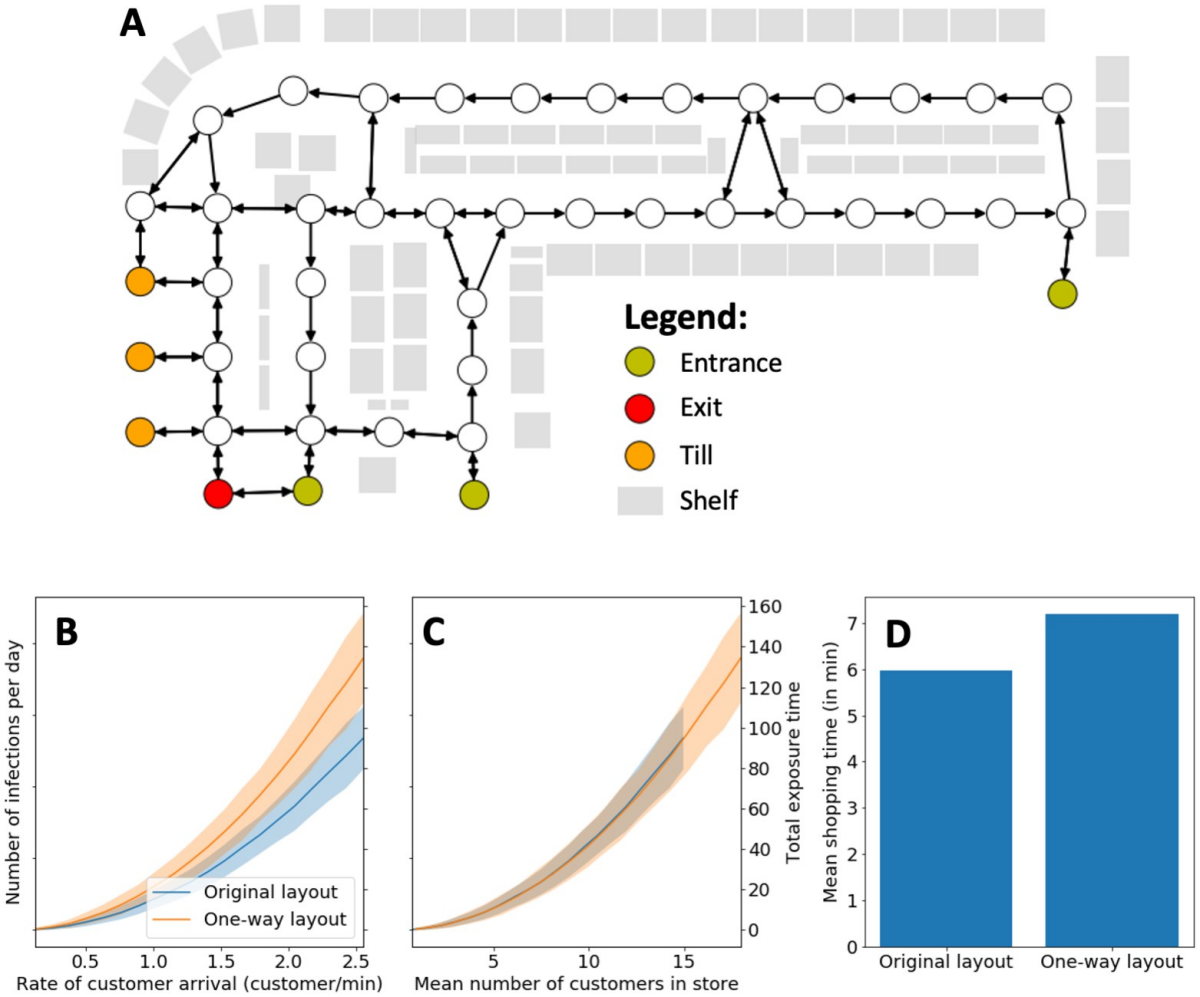
Effect of one-way aisle layout on infections. **(A)** Store layout with one-way aisles. **(B + C)** Number of infections in a store as a function of the customer arrival time and mean number of customers (respectively). We show on the right vertical axis the total exposure time, as the number of infections is proportional to the total exposure time in our model. The one-way layout increases the number of infections with the same arrival rate (see subfigure D). It appears that the number of infections mainly depends on how many customers are in the store on average (see subfigure C). **(D)** Mean customer shopping time. The one-way layout increases the time that customers spend in the store, so more customers are in the store and thereby increase the number of infections.

A number of stores have implemented one-way systems to assist with social distancing and potentially redistributing the flow of customers. We can also assess this policy in our framework by changing the store graph to a directed graph, where some edges are uni-directional. For the synthetic store, we show an example one-way aisle layout in Fig 4A which we call the *one-way store layout*. We need to change the shopping paths in our data, as they may violate the uni-directionality of the one-way store layout. For each path, we first consider again the node sequence (*v*_1_, …, *v*_*K*+3_) from which the path was generated. (As a reminder, *v*_1_ is an entrance node, *v*_*K*+2_ is a till node, *v*_*K*+3_ is an exit node, and the intermediate nodes *v*_2_, …, *v*_*K*+1_ are locations where customers bought one or more items.) The corresponding path for the one-way store layout is then a shortest path that visits each of the nodes *v*_1_, …, *v*_*K*+3_ in sequence in the one-way store layout. In other words, we assume that the one-way layout does not change the order in which customers buy their items; it merely changes the route that they take between items (or between an item and an entrance/till).

According to our model, the one-way layout increases the number of infections (see Fig 4B). To explain this phenomenon, we can look at the mean shopping time between the two layouts. In the one-way store layout, the shopping path necessarily is at least as long as in the original store layout. Therefore, on average, the mean shopping time increases (see Fig 4D), so more customers are in the store at the same time and thus more infections occur. When we fix the mean number of customers in the store, the number of infections is largely the same (see Fig 4C).

### Combination of interventions

We can group the interventions that we listed above as follows:

Group 1: Control the in-flow of customers (by restricting *C*_max_ or λ)
Group 2: Reduction of virus transmissibility (by implementing a face mask policy)
Group 3: Change of store layout (by using a one-way aisle layout)

Interventions between different groups are independent of each other and we can combine interventions from different groups for increased effectiveness. In Table 3, we list a number of possible interventions and some combinations of them. For example, based on our results above, reducing customer arrival rate by 50% leads to 75% fewer infections and 50% smaller chance of infection. However, combining this intervention with a face mask policy, we achieve a 96% in number of infections and 92% reduction in the chance of infections. Practitioners should seek to find the most effective intervention for each group in their stores, and use the combination of interventions that achieve the largest individual reduction in infections or chance in infection for each group.

**Table 3:** Comparison of interventions and combinations of interventions. We compare possible interventions and combinations of interventions by their effect on the number of infections and chance of infection based on our agent-based model. We can achieve the largest amount of reduction by combining the face mask policy with reducing arrival rate or the maximum number of customers.

Intervention	Relative change in number of infections	Relative change in chance of infection
Restrict maximum number of customers *C*_max_ in store to 75% of mean number of customers	-50%	-33%
Reduce customer arrival rate by 50%	-75%	-50%
Face mask policy	-83%	-83%
Face mask policy + reduce arrival rate by 50%	-96%	-92%
Face mask policy + Restrict *C*_max_ to 75% of mean number of customers	-92%	-89%
One-way layout	+42%	+42%

### Limitations

Our model comes with a number of limitations. Firstly, a customer’s path in our model does not depend on other customers, whereas we expect a customer’s path to change depending on, for example, the crowdedness of other zones. Unlike in [16], our spatial topology is more coarsely grained, where customers are either in the same zone or not, and we therefore do not take the precise distance between customers into account. We also assume that the chance of infection is proportional to the exposure time, whereas in reality it may be non-linear (e.g., represented by a logistic function to model infectious dosage). In our simulations, we used a constant arrival rate and random shopping paths that do not change with time, while we expect time-varying arrival rates and shopping path distributions in reality. However, it is relatively straightforward to incorporate modifications to the transmission function or to the arrival rate into our agent-based model. Lastly, all results that we present in this article are from simulations on a synthetic data set using a transmission rate *β* which was estimated in a different setting. Therefore, we anticipate our point estimate for *β* may not be very accurate, and the concrete results that we presented may have limited generalisation power. The number of infections of store that we estimated should therefore not be taken at face value. Nonetheless, even without an accurate measure of *β*, we anticipate that the exposure time is a relevant metric to measure the relative risk of transmission. Validation of our results is likely to be difficult, as no data on the number of infections in supermarkets exists. A possible way to estimate this is to survey people who have gotten infected and ask where they likely got infected. While this will likely add some biases, such data could be helpful. The closest such data to the best of our knowledge is the survey data by Public Health England on the percentage of infected people who have visited a supermarket prior to getting a positive COVID-19 test [33]. However, one cannot infer from this data directly how many of those people were infected in the supermarket.

## Conclusion

We presented a model for modelling virus transmission (in particular, SARS-CoV-2 transmissions, which causes COVID-19, but it is more generally applicable) in supermarkets based on an agent-based model of customers traversing from zone to zone and being exposed to potential virus infection when in the same zone as an infected customer. We measured the risk of virus transmission by the total time that susceptible customers spent in the same zone as infected customers (and called this time the exposure time). We demonstrated the capabilities of the model by applying it to synthetic data with model parameters specific to SARS-CoV-2. We showed how one can use the model to identify hotspots and bottlenecks, estimate the reduction in virus transmissions in different scenarios such as restricting the maximum number of customers in a store or implementing a one-way aisle system. In particular, in our synthetic store, a directed store layout does not help in reducing the exposure time, as it increases the customer shopping time, so more customers are in the store at any given time. The best policy among those that we tested is to restrict the arrival rate of customers or the maximum number of customers together with a mandatory face mask policy; doing so can significantly reduce the number of infections and the chance of getting infected in a supermarket. We invite retailers to use our model to identify bottlenecks that lead to crowded zones as well as to inform them on the best store policy. In future work, it would be interesting to understand which store network features or size of stores lead to lower number of infections and lower chance of infection.

## Supporting information

S1 Fig Sensitivity analysis. We plot the mean number of infections and chance of infection (with the shaded area showing the standard deviation) as a function of **(A) + (D)** traversal time *τ*, **(B) + (E)** proportion *p*_*I*_ of infectious customers, and **(C) + (F)** transmission parameter *β*. As the number of infections is a linear function of the total exposure time, we also show the total exposure time on the right vertical axis in subfigures (A)–(C). Similarly, we show the mean exposure time on the right vertical axis in subfigures (D)–(F).
(TIF)

S1 Appendix (PDF)
