# Supplementary figures and images for: Modelling COVID-19 transmission in supermarkets using an agent-based model
Source: PLoS One. 2021 Apr 9;16(4):e0249821. doi: 10.1371/journal.pone.0249821 (PMC8034715; doi:10.1371/journal.pone.0249821)

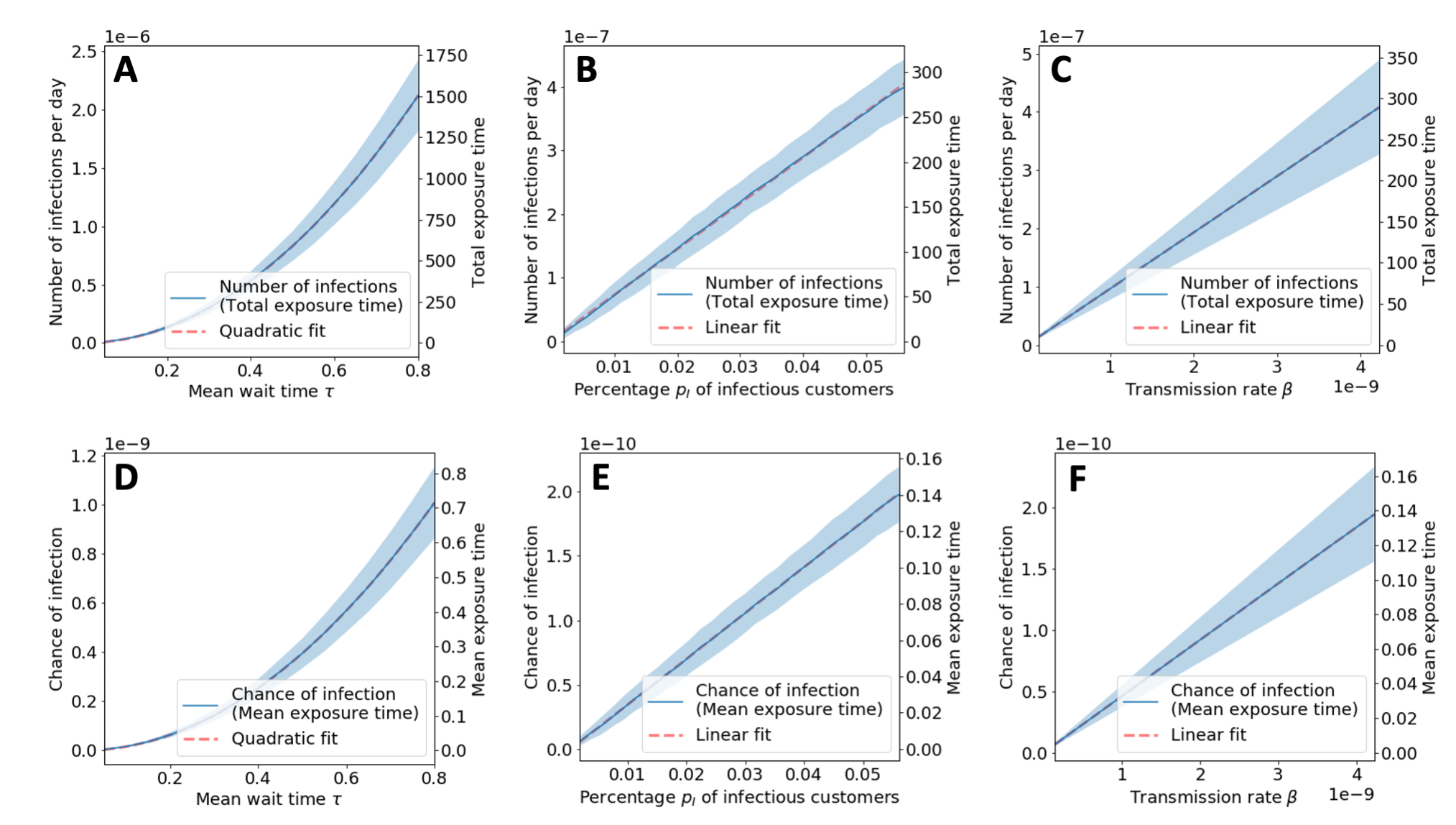

Supplement: S1 Fig — We plot the mean number of infections and chance of infection (with the shaded area showing the standard deviation) as a function of (A) + (D) traversal time τ, (B) + (E) proportion pI of infectious customers, and (C) + (F) transmission parameter β. As the number of infections is a linear function of the total exposure time, we also show the total exposure time on the right vertical axis in subfigures (A)–(C). Similarly, we show the mean exposure time on the right vertical axis in subfigures (D)–(F). (TIF) [file pone.0249821.s001.tif]
