# Supplementary material for: Modelling COVID-19 transmission in supermarkets using an agent-based model
Source: PLoS One. 2021 Apr 9;16(4):e0249821. doi: 10.1371/journal.pone.0249821 (PMC8034715; doi:10.1371/journal.pone.0249821)
Supplement: S1 Appendix — (PDF) [file pone.0249821.s002.pdf]

Modelling COVID-19 transmission in supermarkets using an agent-based model

Fabian Ying [1], Neave O'Clery[2*],

1 Quantitative Research, G-Research, London, UK

2 Centre for Advanced Spatial Analysis, UCL, London, UK

* n.oclery@ucl.ac.uk

Supporting information

S1 Fig. Sensitivity analysis. We plot the mean number of infections and chance of infection (with the shaded area showing the standard deviation) as a function of **(A) + (D)** traversal time τ, **(B) + (E)** proportion p_I of infectious customers, and **(C) + (F)** transmission parameter β. As the number of infections is a linear function of the total exposure time, we also show the total exposure time on the right vertical axis in subfigures (A) - (C). Similarly, we show the mean exposure time on the right vertical axis in subfigures (D) - (F).

S1 Appendix. Sensitivity analysis. In this section, we explore further how the number of infections per day and chance of infection depends on our model parameters.

We see that the number of infections and chance of infection increases quadratically with the mean wait time τ at each node, linearly with the proportion p_I of infectious customers and the transmission rate β (see S1 Fig). We can explain the quadratic relationship between the number of infections and the mean wait time τ in the same way as when we varied the arrival rate: the number of customers in the store increases linearly with τ and the number of infections increases quadratically with the number of customers. Unlike when we varied arrival rate, the chance of infection does increase linearly but quadratically with τ. This is because we have not only more customers

(and thus infectious customers) in the store, but each customer stays also longer in the store, thus yielding a quadratic scaling.
